# Seasonal Dynamics Versus Vertical Stratification of Mosquitoes (Diptera: Culicidae) in an Atlantic Forest Remnant, Brazil: A Focus on the Mansoniini Tribe

**DOI:** 10.3390/tropicalmed11020039

**Published:** 2026-01-30

**Authors:** Cecília Ferreira de Mello, Wellington Thadeu de Alcantara Azevedo, Shayenne Olsson Freitas Silva, Samara Campos Alves, Jeronimo Alencar

**Affiliations:** 1 Laboratório Diptera, Instituto Oswaldo Cruz (Fiocruz), Avenida Brasil 4365, Manguinhos, Rio de Janeiro 21040-360, RJ, Brazil; cecilia.mello@ioc.fiocruz.br (C.F.d.M.); samaracampos255@gmail.com (S.C.A.); 2 Departamento de Microbiologia e Parasitologia, Instituto Biomédico, Centro de Ciências Biológicas e da Saúde, Universidade Federal do Estado do Rio de Janeiro, Avenida Pasteur, 458, Urca, Rio de Janeiro 22290-240, RJ, Brazil; wellingtontaa@yahoo.com.br; 3 Faculdade de Educação, Campus Universitário Darcy Ribeiro, Universidade de Brasília, Prédio FE 03—Sala BT 06/14, Asa Norte, Brasília 70.910-900, DF, Brazil; shayenneolsson@gmail.com

**Keywords:** community ecology, seasonal dynamics, *Mansonia*, *Coquillettidia*, vector ecology, Atlantic Forest

## Abstract

Mosquitoes (Diptera: Culicidae) exhibit vertical stratification patterns in forest environments, a fundamental ecological aspect for understanding niche occupation patterns, host-seeking behavior, and consequently arbovirus transmission mechanisms. Despite the relevance of this topic, available studies mostly focus on genera such as *Aedes*, *Haemagogus*, and *Sabethes* which are traditionally associated with arbovirus transmission. There are still important gaps regarding stratification and seasonality in the Mansoniini tribe, whose biology and epidemiological role remain underexplored, especially in highly biodiverse ecosystems such as the Atlantic Forest. This study evaluated the influence of seasonality and vertical stratification on the mosquito community, with a detailed focus on the Mansoniini tribe, in an Atlantic Forest fragment in Brazil, between May 2023 and December 2024. Captures were performed monthly using CDC light traps positioned at 1.5 m and 10 m heights, and specimens were morphologically identified. A total of 880 mosquitoes from nine genera and 24 species were captured, of which 91 (10.3%) belonged to the Mansoniini tribe. The most abundant species were *Coquillettidia fasciolata* and *Mansonia titillans*, recorded in both strata. Our results indicate no marked vertical segregation for the studied mosquito community in this specific location, but a strong influence of seasonality, particularly for the Mansoniini tribe, reinforcing the role of meteorological data on the population structure of these species. These site-specific findings offer a foundational ecological portrait and a robust methodological template for a neglected taxon. They generate critical, testable hypotheses about niche partitioning in fragmented forests and underscore the necessity for broader spatial replication to disentangle the relative influence of seasonal versus vertical drivers in similar ecosystems.

## 1. Introduction

The spatial distribution of mosquitoes (Diptera: Culicidae) in forest environments is strongly influenced by microclimatic factors, host availability, and vegetation characteristics [1]. Among the various aspects of vector ecology, vertical stratification (the variation in abundance and species composition at different heights in the forest) has proven to be a key element in understanding flight behavior, the dynamics of arbovirus transmission, and host selection by different species [2,3,4,5].

Classical and recent studies indicate that mosquito flight height may be associated with environmental and biological variables, such as host height, relative humidity, temperature, light intensity and quality, as well as vegetation structure and stratification. In parallel, factors such as the availability of larval habitats, the presence of physical barriers in the environment, and predator pressure can also influence the vertical dispersal patterns of these insects [6,7,8].

The influence of vertical stratification on mosquito ecology has been recognized since the pioneering work of Deane, Damasceno, Arouck [9,10], who highlighted the role of height in interactions with arboreal hosts and in the transmission dynamics of arboviruses in tropical environments. Later, Forattini [11] systematized these patterns, describing distinct vertical behaviors among genera such as *Haemagogus*, *Sabethes* and *Culex*.

Although this approach has already been explored for some medically important genera, studies focusing on the preferred flight height of mosquitoes of the Mansoniini tribe, especially the *Mansonia* and *Coquillettidia* genera, remain scarce. This gap is particularly relevant in tropical regions, such as the Atlantic Forest, where the structural complexity of the forest and microclimatic gradients can influence the vertical behavior of these insects. Studies in forest remnants show that *Mansonia* species are most active in the lower stratum, close to 1.5 m above the ground, highlighting the need for more detailed investigations into the vertical stratification of these mosquitoes in tropical environments [12].

The Mansoniini tribe comprises approximately 83 species distributed across two genera, *Mansonia* (25 species) and *Coquillettidia* (58 species), both belonging to the subfamily Culicinae. They are widely distributed throughout tropical regions, particularly in South America [13].

The species in this group exhibit unique biological and behavioral characteristics, such as the use of aquatic plants as a respiratory substrate during the larval stage and preferentially crepuscular and nocturnal hematophagic habits [14,15,16,17]. These ecological peculiarities, along with the potential role of some species in the transmission of arboviruses such as Venezuelan Equine Encephalitis (VEEV) and Mayaro (MAYV) [18,19,20,21,22,23,24], suggest a strong association with specific forest environments, with vertical stratification being a possible ecological factor regulating their activity. Despite this potential epidemiological relevance, the ecology of the tribe, including its response to seasonal and vertical gradients compared to more common vector genera, remains poorly understood.

Although the importance of vertical stratification in mosquito ecology has been widely recognized, available information primarily focuses on wild vectors of arboviruses such as yellow fever and dengue in high transmission areas [19].

Although some species of the Mansoniini tribe have already been associated with the transmission of arboviruses such as Venezuelan Equine Encephalitis virus (VEEV), Eastern Equine Encephalitis virus (EEEV), and Mayaro virus (MAYV), their potential role in arbovirus ecology is still underestimated, in part due to the scarcity of data on their behavioral patterns and distribution [18,19,20,21,22,23,24].

Given this scenario, this study aimed to investigate the influence of vertical stratification and seasonality on the mosquito community in an Atlantic Forest fragment, with a specific focus on the Mansoniini tribe. A secondary objective was to compare the ecological responses (vertical distribution and seasonal variation) of Mansoniini with those of other abundant mosquito genera (*Culex* and *Aedes*) captured under the same conditions in order to contextualize the tribe’s patterns within the local culicid fauna.

## 2. Method

### 2.1. Ethics Statement

All research was carried out in accordance with scientific license number 84,318 provided by SISBIO/IBAMA (Authorization and Information System on Biodiversity/Brazilian Institute of Environment and Renewable Natural Resources) for the capture of culicids throughout the national territory.

### 2.2. Study Area

**Figure 1:**
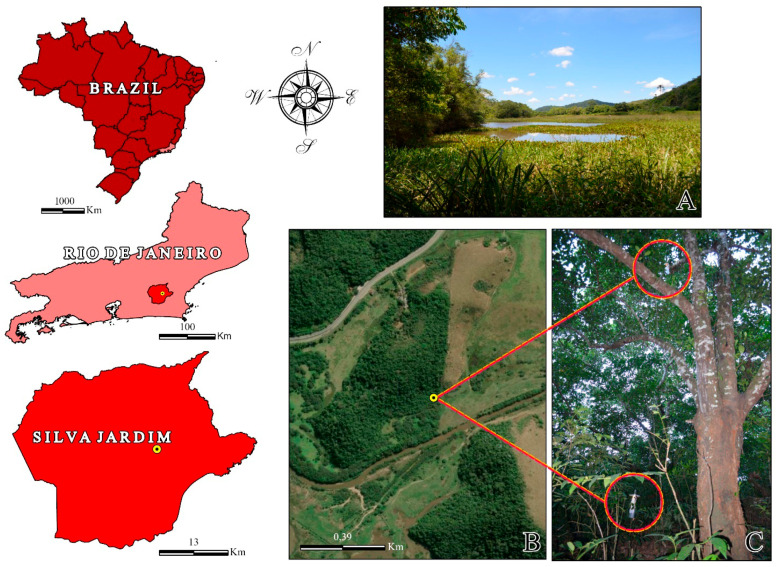
Location and characterization of the study area at the Sítio Recanto Preservar, municipality of Silva Jardim, state of Rio de Janeiro, Brazil. (**A**) Panoramic view of the wetland in the study area. (**B**) Satellite image highlighting the collection area. (**C**) Tree located near the wetland, where traps were placed at 1.5 m and 10 m heights.

The study was conducted in an Atlantic Forest fragment located at the Sítio Recanto Preservar, municipality of Silva Jardim, state of Rio de Janeiro, Brazil (22°34′00.1″ S 42°21′33.2″ W) (Figure 1). The study area is connected to the Poço das Antas Biological Reserve, an important federal conservation unit. According to the Köppen classification (1948), the local climate is a humid tropical type As, strongly influenced by the Atlantic Ocean and characterized by high temperatures and elevated humidity throughout the year. The rainy season occurs from November to February, whereas the dry season predominates from June to September. Despite higher precipitation during the summer, intra-seasonal dry spells may occur, with interruptions of up to two to three weeks in rainfall and temperatures frequently exceeding 38–40 °C. The hydrological regime of the area is determined by the combined influence of direct precipitation and the São João River Basin [25]. The environment is characterized by secondary vegetation in different regeneration stages and the presence of the São João River, which runs through the property. The predominant vegetation at the site includes native Atlantic Forest species, as well as exotic species such as jackfruit (*Artocarpus heterophyllus*), used as support for the traps [26].

### 2.3. Mosquito Sampling and Identification

Captures were conducted monthly between May 2023 and December 2024, totaling 20 campaigns. CDC traps were installed at two vertical levels, 1.5 m (understory) and 10 m (canopy), both positioned on the same jackfruit tree. The use of a single tree for both traps represents a study limitation regarding spatial replication, and results concerning vertical stratification should be interpreted as preliminary observations for this specific point. The traps were set at 5:00 PM and retrieved at 8:00 AM the following day, operating continuously for 15 h.

### 2.4. Morphological Identification

Species identification was performed based on direct observation of morphological characteristics evident under a stereomicroscope and consultation of species descriptions/diagnoses using dichotomous keys developed by Lane [27]; Consoli, Oliveira [28]; Forattini [11]; Assumpção [29]; and Barbosa [30]. The abbreviation of genera and subgenera followed the standards established for the Reinert [31] group. All specimens have been catalogued and are awaiting assignment of voucher numbers by the Oswaldo Cruz Institute Entomological Collection (Fiocruz) under the title “*Coleção Mata Atlântica, Recanto Preservar*”. Once confirmed, these numbers will be made publicly available and included in the Supplementary Materials, ensuring long-term preservation, traceability, and accessibility for future research.

Meteorological variables, such as temperature, relative humidity, atmospheric pressure, wind direction and speed, were obtained using a wireless digital weather station (Instrutemp ITWT2000 model, Instrumentos de Medição, São Paulo, Brazil). Daily averages for each variable were calculated for the 7-day period preceding each monthly capture in order to record the relevant conditions of adult mosquito activity. These data were later used as auxiliary environmental variables in the analysis of species occurrence and abundance throughout the sampling period.

### 2.5. Georeferencing

ArcGIS Pro 3.0.1 (Esri, Redlands, CA, USA) software was used to create the location map of the collection areas. The geographic coordinates of the sampling points were entered into the program, enabling generation of a thematic map which spatially represents the studied location and contributing to accurate geographic visualization of the field data.

### 2.6. Statistical Analyses

The data obtained were tabulated in an Excel spreadsheet for analysis using the PAST (Paleontological Statistics Software, version 4.05) and RStudio (RStudio, PBC, Boston, MA, USA, version 2025.05.1+513) statistical programs, adopting a significance level of 5% (*p* < 0.05). A Coleman’s species accumulation curve was constructed to evaluate the sampling effort. The influence of trap height and preference of Mansoniini dipterans for a seasonal period (dry or rainy) were evaluated through PERMANOVA (Permutational Multivariate Analysis of Variance) with 999 permutations. In turn, SIMPER (Similarity Percentage Analysis) was applied to identify the species which most contributed to the dissimilarities between heights and seasons. The functions used were adonis2 and simper, both from the vegan package in the R software program (version 2025.09.2). The indval (Indicator Value Analysis, IndVal) function from the labdsv package was used to verify species specificity at a given time or season. Species diversity at each time and season was estimated using the Shannon-Wiener diversity index (H’), and evenness was assessed using the Pielou’s evenness index (J’). The similarity of faunal composition between strata was analyzed using the Jaccard similarity index. A Bray–Curtis dissimilarity plot was produced to illustrate the similarities between the months.

Generalized Linear Models (GLMs) with a negative binomial distribution (to account for overdispersion in count data) were applied to enable a comparative ecological analysis using the glm.nb function form the MASS package in R. Separate models were constructed for the total abundance of (a) the Mansoniini tribe, (b) the genus *Culex*, (c) the *Aedes* genus. Each model tested the fixed effects of ‘Height’ (1.5 m, 10 m) and ‘Season’ (Dry, Rainy), as well as their interaction. Models were fitted using a log link function, ensuring positive fitted values for abundance. Model selection was based on AIC, and goodness-of-fit was assessed using DHARMa residual diagnostics and were considered adequate. This approach enabled a direct comparison of how these key taxa responded to the same vertical and seasonal gradients.

## 3. Results

A total of 880 individuals belonging to nine genera and 24 species of Culicidae were captured. The samples included representatives of the genera: *Aedeomyia*, *Aedes*, *Anopheles*, *Coquillettidia*, *Culex*, *Mansonia*, *Orthopodomyia*, *Psorophora* and *Uranotaenia*. Among all recorded species, *Culex* (*Melanoconion*) sp. was the most abundant taxon, with 314 individuals (35.68% of the total), followed by *Aedes scapularis* (Rondani, 1848) (n = 138; 15.68%) and *Culex* (*Culex*) sp. (n = 98; 11.14%) (Table 1).

**Figure 2:**
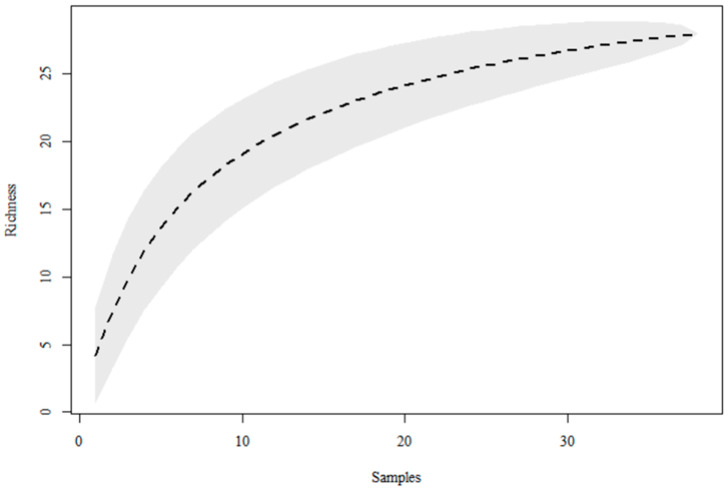
Coleman’s species accumulation curve demonstrating the sufficiency of the sampling effort of the survey of mosquitoes (Diptera: Culicidae) collected in CDC traps at the Sítio Recanto Preservar, Silva Jardim, Rio de Janeiro, Brazil, between May 2023 and December 2024.

A total of 91 specimens of the Mansoniini tribe were captured, representing 10.3% of the family. The most abundant species were *Coquillettidia fasciolata* (Lynch Arribálzaga, 1891), representing 3.7% of the specimens (n = 33), and *Mansonia titillans* (Walker, 1848), which comprised 2.9% of the sampled population. The species with the lowest abundance was *Mansonia humeralis* Dyar & Knab, 1916, with only 2 individuals, representing 0.2% of the population. According to Coleman’s accumulation curve, the sampling effort employed in the study was sufficient to sample the populations of the Mansoniini tribe at the study site, since the curve approached an asymptote (Figure 2).

**Figure 3:**
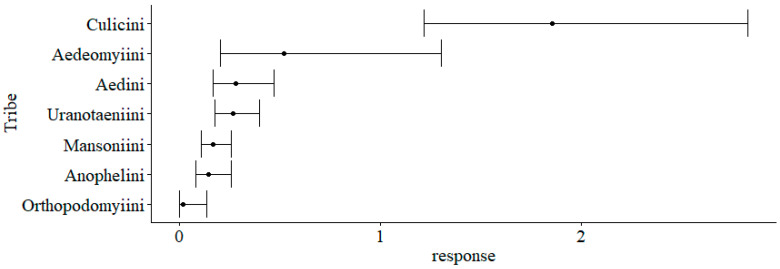
Confidence intervals (95%) and estimated marginal for the total abundance of mosquitoes (Diptera: Culicidae) collected in CDC traps at the Sítio Recanto Preservar, Silva Jardim, Rio de Janeiro, Brazil, between May 2023 and December 2024.

The GLM comparing the abundances of different taxa revealed that Culicini was the most abundant tribe (Table 2). It showed 3.6 times greater abundance (*p* = 0.013) compared to the Aedeomyiini tribe, while the Aedini and Uranotaeniini tribes did not differ from the reference. Anophelini, Mansoniini, and Orthopodomyiini were less abundant than Aedeomyiini (*p* < 0.05) (Figure 3).

**Figure 4:**
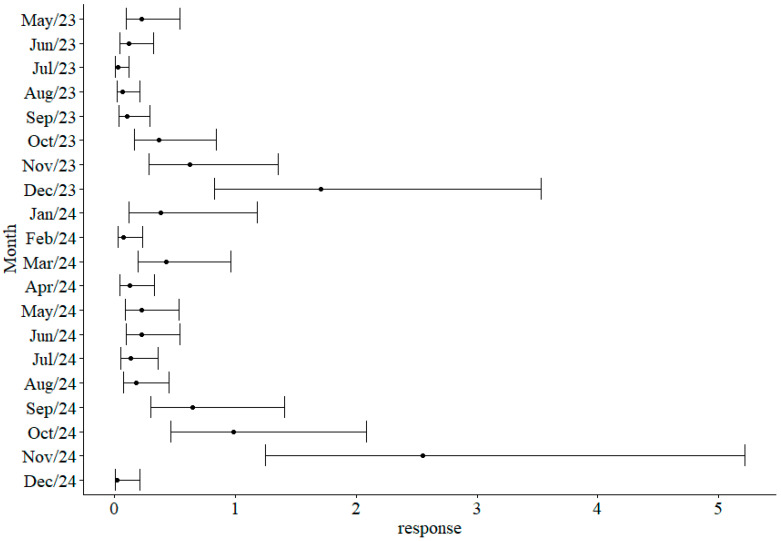
Confidence intervals (95%) and estimated marginal for the monthly abundance of mosquitoes (Diptera: Culicidae) collected in CDC traps at the Sítio Recanto Preservar, Silva Jardim, Rio de Janeiro, Brazil, between May 2023 and December 2024.

The season was also relevant according to the GLM (Table 2), with greater abundance mainly observed during the rainy season (November–March). When compared to the period of April 2024, greater abundance was observed during March, September, October and November of the same year, as well as November and December of 2023 (Figure 4).

### Seasonal Variation in the Mansoniini Tribe

Table 3 below shows the abundance variation for each species regarding their preference for the dry and rainy seasonal periods characteristic of the studied region.

**Figure 5:**
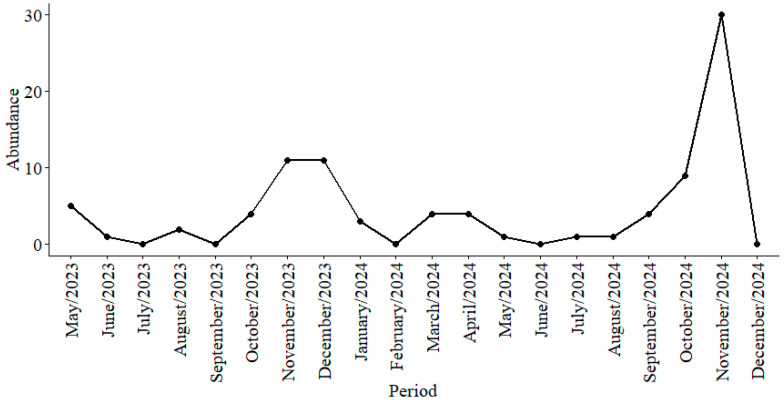
Monthly abundance of Mansoniini tribe species captured using CDC traps at the Sítio Recanto Preservar, Silva Jardim, Rio de Janeiro, Brazil, between May 2023 and December 2024.

The GLM analysis comparing the abundance of the Mansoniini tribe showed that *Cq. humeralis* differs in abundance from *Cq. chrysonotum*, and revealed a significant effect of the season (Table 4). The additional environmental variables did not show additional effects, which suggest the season explains the main environmental variation associated with abundance, and encompass environmental variations within seasons. The Mansoniini tribe showed its highest peak abundance in November 2024 (n = 30), followed by November and December 2023 (n = 11), corresponding to the rainy seasons (Figure 5).

This difference is highlighted by the PERMANOVA analysis showing a difference in species composition between the studied seasonal periods (F = 2.254, R^2^ = 0.111, *p* = 0.047). Abundance was higher in the rainy period, especially for *Ma. tillitans*, *Cq. fasciolata* and *Cq. chrysonotum*. The capture was generally higher during this period (n = 59), with *Cq. fasciolata* occurring in greater abundance (n = 20). Only 32 individuals were captured during the dry periods, with *Cq. fasciolata* also being the most abundant species (n = 13) (Table 3). The indicator analysis (indval) confirmed the specificity of *Cq. venezuelensis* with the rainy period (indval = 0.375, *p* = 0.044).

**Figure 6:**
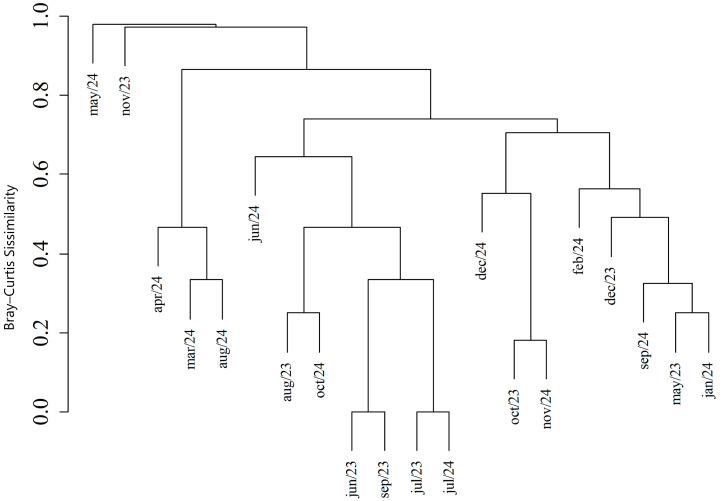
Monthly similarity based on the Bray–Curtis index in the composition of the Mansoniini tribe collected using CDC traps at the Sítio Recanto Preservar, Silva Jardim, Rio de Janeiro, between May 2023 and December 2024 based on the Bray–Curtis index.

Diversity analysis between seasonal periods revealed greater diversity during the rainy season (H’ = 1.566) compared to the dry season (H’ = 1.341), as well as greater evenness (J’ = 0.874) (Table 3). The similarity between the two periods was 0.429 according to the Jaccard similarity index. A cluster analysis was performed using the Bray–Curtis Similarity Index to illustrate the similarity between mosquito communities by month. It was observed that the most similar months were October 2023 and November 2024 (BC = 0.82), with the co-occurrence of *Cq. chrysonotum*, *Cq. fasciolata* and *Ma. titillans*; May 2023 and January 2024 (BC = 0.75), with *Cq. fasciolata* in equal abundance; and August 2023 and October 2024 (BC = 0.75), with the co-occurrence of *Cq. fasciolata* and *Ma. titillans*. The grouping of June, July and September 2023 and July 2024 with high similarity (BC = 1) occurred due to the low capture in these months (n = 0 or 1) (Figure 6).

## 4. Discussion

This study provides the first focused assessment of vertical stratification and seasonality of the Mansoniini tribe in an Atlantic Forest fragment, expanding knowledge about the ecology of this group in one of the most biodiverse and threatened biomes on the planet. Furthermore, it contextualizes the tribe’s ecological responses within the local mosquito community by comparing these patterns with those of other dominant genera.

High abundance of *Culex* (*Melanoconion*) sp. and *Aedes scapularis* stood out among all the Culicidae recorded during the study period. The ability of *Ae. scapularis* to occupy different environments was characterized by Forattini, Gomes, Natal, Kakitani, Marucci [32], who recorded its occurrence from forest areas to peri-urban environments, with home penetration associated with the search for hosts from different groups. In the same study, *Culex* (*Melanoconion*) sp. was observed in both forest fragments and modified areas, suggesting adaptability and a potential role in arbovirus transmission. These patterns are consistent with the predominance observed in this study and suggest that both species exhibit high resilience and colonization capacity in fragmented Atlantic Forest landscapes.

The main finding of this study was the predominance of seasonal dynamics over vertical stratification in structuring the mosquito community at the sampled point. Although the total abundance of Culicidae was higher in traps installed at 10 m, analysis of the species composition of the Mansoniini tribe did not indicate significant differences in the species group between the understory (1.5 m) and the canopy (10 m). This absence of significant vertical stratification was corroborated by the GLM analysis, which showed no effect of height on the abundance of Mansoniini, *Culex*, or *Aedes*. This suggests that the studied mosquito community does not exhibit marked vertical segregation under the specific conditions of this fragment (i.e., reduced vertical microclimatic gradient, proximity to larval habitats). It is crucial to highlight that this conclusion is based on a single sampling point (one tree), constituting a limitation in spatial replication. Therefore, the observed non-vertical stratification pattern should be interpreted as preliminary and specific to the local conditions of this study, requiring validation through future research with spatial replication across multiple trees or transects. This overlap pattern between strata was also recorded by Tissot, Navarro-Silva [12] in an Atlantic Forest remnant in Paraná, where species such as *Ma. titillans* and *Cq. venezuelensis* occurred at both levels. Similarly, Confalonieri and Costa Neto [33] reported that several mosquito species utilize different vegetation heights flexibly, likely due to factors such as host availability and microclimate. On the other hand, studies conducted in the Amazon [7,19] indicate more pronounced vertical segregation for some groups, suggesting that this pattern may vary between biomes due to differences in forest structure and fauna composition.

The equivalent use of both strata by *Cq. fasciolata* and *Ma. titillans* suggests versatile flight behavior. A similar pattern had previously been observed in an Atlantic Forest remnant by Tissot, Navarro-Silva [12], reinforcing the idea of broad vertical use within the tribe. Confalonieri, Costa Neto [33] also documented plasticity in Culicidae with generalist foraging habits, which may represent an adaptive advantage. In the context of the present study, this behavior may be favored by the slight microclimatic gradient between the understory and the canopy, which reduces barriers to vertical movement and allows species to reach different heights to explore available hosts and microhabitats.

In contrast to the vertical dimension, seasonality emerged as a strong structuring factor, especially for the Mansoniini tribe. The species composition of the Mansoniini tribe differed significantly between the dry and rainy seasons, with greater abundance, richness, and diversity observed in the rainy season. A similar pattern was also observed in the Central Amazon, where de Mello, Figueiró, Roque, Maia, da Costa Ferreira, Guimarães, Alencar [34] identified accumulated precipitation and maximum temperature as determining factors for the Mansoniini community structure. Despite the structural differences between the Amazon rainforest and the Atlantic Forest, the seasonal pattern appears consistent, suggesting that the greater availability of aquatic breeding sites during the wet season is a key factor in the population dynamics of these species. In the present study, *Cq. venezuelensis* and *Ma. pseudotitillans* exclusively occurred in the rainy season, indicating a strong association with humid microhabitats, as supported by indicator analysis (IndVal). This pattern is consistent with evidence that *Mansonia* spp. benefits from high water level conditions and increased availability of aquatic macrophytes during the flood season Ferreira, Pereira, Har, Hamada [35].

Saraiva, Furtado, Maitra, Carvalho, Galardo, Lima [36] observed that seasonal patterns of *Mansonia* spp. in Porto Velho, Rondônia, were strongly associated with meteorological variables related to relative humidity. However, it was observed in the present study that the species were more abundant in the rainy period, whereas relative humidity was not significant.

The observed predominance of seasonal variation, the absence of vertical stratification at the sampled point, and the comparative analysis of the Mansoniini tribe species provide relevant information about the ecology of these mosquitoes in Atlantic Forest fragments. The tribe’s marked seasonality underscores its tight coupling with rainy periods and the availability of aquatic habitats for immature stages. The absence of pronounced vertical segregation indicates that individuals can use both sampled strata, which is consistent with studies documenting behavioral flexibility in Mansoniini [5,7].

The greater diversity and abundance during the rainy season highlight the fundamental role of larval habitat availability and moisture conditions in the population dynamics of these species, an aspect widely reported for humid tropical environments [17]. The equivalent use of different strata by various species also suggests tolerance of communities to structural habitat variations, as observed in fragmented environments [19,33], although this finding requires confirmation through studies with robust spatial designs. Future studies which integrate trophic analyses, detailed assessment of microhabitats, spatial replication across multiple points, and temporal activity patterns may deepen our understanding of the ecological and epidemiological role of *Mansoniini* in Atlantic Forest fragments.

## Figures and Tables

**Table 1:** Abundance of mosquitoes (Diptera: Culicidae) collected using CDC traps at two heights (1.5 and 10 m) at the Sítio Recanto Preservar, Silva Jardim, Rio de Janeiro, Brazil, between May 2023 and December 2024.

Species	Total					
	1.5		10			
	n	%	n	%	n	%
**Aedeomyiini**						
*Aedeomiya squamipennis* (Lynch Arribálzaga, 1878)	11	3.1	37	7.0	48	5.5
**Aedini**						
*Aedes rhyacophilus* da Costa Lima, 1933	16	4.6	24	4.5	40	4.5
*Aedes scapularis* (Rondani, 1848)	43	12.3	95	18.0	138	15.7
*Aedes serratus* S.l (Theobald, 1901)	1	0.3	4	0.8	5	0.6
*Psorophora ferox* (Humboldt, 1819)	-	-	3	0.6	3	0.3
**Anophelini**						
*Anopheles evansae* (Brèthes, 1926)	3	0.9	7	1.3	10	1.1
*Anopheles kompi* Edwards, 1930	-	-	3	0.6	3	0.3
*Anopheles triannulatus* (Neiva & Pinto, 1922)	4	1.1	16	3.0	20	2.3
**Culicini**						
*Culex (Culex)* sp.	26	7.4	72	13.6	98	11.1
*Culex (Melanoconion)* sp.	150	42.7	164	31.0	314	35.7
*Culex (Microculex) davisi*	1	0.3	-	-	1	0.1
*Culex (Microculex) pleuristriatus*	1	0.3	-	-	1	0.1
**Mansoniini**						
*Coquillettidia chrysonotum* (Peryassú, 1922)	7	2.0	7	1.3	14	1.6
*Coquillettidia fasciolata* (Lynch Arribálzaga, 1891)	16	4.6	17	3.2	33	3.8
*Coquillettidia venezuelensis* (Theobald, 1912) (in Surcouf, 1912)	4	1.1	4	0.8	8	0.9
*Mansonia humeralis* Dyar & Knab, 1916	2	0.6	-	-	2	0.2
*Mansonia indubitans* Dyar & Shannon, 1925	1	0.3	3	0.6	4	0.5
*Mansonia pseudotitillans* (Theobald, 1901)	3	0.9	1	0.2	4	0.5
*Mansonia titillans* (Walker, 1848)	8	2.3	18	3.4	26	3.0
**Orthopodomyiini**						
*Orthopodomyia albicosta* (Lutz, 1904) (in Bourroul, 1904)	-	-	1	0.2	1	0.1
**Uranotaeniini**						
*Uranotaenia apicalis* Theobald, 1903	13	3.7	5	0.9	18	2.0
*Uranotaenia briseis* Dyar 1925	2	0.6	3	0.6	5	0.6
*Uranotaenia geometrica* Theobald, 1901	7	2.0	13	2.5	20	2.3
*Uranotaenia hystera* Dyar & Knab, 1913	16	4.6	13	2.5	29	3.3
*Uranotaenia lowii* Theobald, 1901	9	2.6	12	2.3	21	2.4
*Uranotaenia pulcherrima* Lynch Arribálzaga, 1891	7	2.0	5	0.9	12	1.4
*Uranotaenia* sp.	-	-	2	0.4	2	0.2
**Total**	**351**	**100**	**529**	**100**	**880**	**100**

**Table 2:** Parameters and *p*-values from the Generalized Linear Model results with negative binomial distribution explaining the abundance of Culicidae tribes collected at the Sítio Recanto Preservar, municipality of Silva Jardim, state of Rio de Janeiro, Brazil, between May 2023 and December 2024. Predicted variables: tribe, period, and height.

	Estimate	Std. Error	z-Value	Pr (>|z|)	Significance
(Intercept)	−1.27	0.67	−1.9	0.058	
Aedini	−0.61	0.53	−1.1	0.250	
Tribe: Anophelini ^1^	−1.27	0.55	−2.3	0.021	*
Tribe: Culicini ^1^	1.27	0.51	2.5	0.013	*
Tribe: Mansoniini ^1^	−1.13	0.51	−2.2	0.027	*
Tribe: Orthopodomyiini ^1^	−3.37	1.13	−3.0	0.003	**
Tribe: Uranotaeniini	−0.66	0.51	−1.3	0.189	
Period: Aug/23	−0.62	0.74	−0.8	0.397	
Period: Aug/24	0.39	0.65	0.6	0.554	
Period: Dec/23 ^2^	2.63	0.59	4.5	<0.001	***
Period: Dec/24	−1.62	1.19	−1.4	0.173	
Period: Feb/24	−0.48	0.72	−0.7	0.502	
Period: Jan/24	1.12	0.74	1.5	0.128	
Period: Jul/23	−1.56	0.89	−1.8	0.080	
Period: Jul/24	0.10	0.67	0.1	0.886	
Period: Jun/23	−0.04	0.68	−0.1	0.949	
Period: Jun/24	0.60	0.64	0.9	0.344	
Period: Mar/24 ^2^	1.25	0.61	2.0	0.041	*
Period: May/23	0.60	0.64	0.9	0.344	
Period: May/24	0.59	0.64	0.9	0.358	
Period: Nov/23 ^2^	1.62	0.60	2.7	0.007	**
Period: Nov/24 ^2^	3.03	0.59	5.2	<0.001	***
Period: Oct/23	1.10	0.62	1.8	0.075	
Period: Oct/24 ^2^	2.08	0.60	3.5	<0.001	***
Period: Sep/23	−0.16	0.69	−0.2	0.811	
Period: Sep/24 ^2^	1.66	0.60	2.8	0.006	**
Height: 10 m	−0.01	0.20	0.0	0.974	

^1^ Difference from *Coquillettidia chrysonotum*. ^2^ Difference from Dry Season. Signif. codes: ‘***’ (3 asterisks): 0.001; ‘**’ (2 asterisks): 0.01; ‘*’ (1 asterisk): 0.05.

**Table 3:** Spatial distribution and diversity indices of species of the Mansoniini tribe captured using CDC traps in two seasonal periods (dry and rainy) at the Sítio Recanto Preservar, Silva Jardim, Rio de Janeiro, Brazil, between May 2023 and December 2024.

Species	Season			
	Dry		Rainy	
	n	%	n	%
*Coquillettidia chrysonotum*	8	25.0	6	10.2
*Coquillettidia fasciolata*	13	40.6	20	33.9
*Coquillettidia venezuelensis*	-	-	8	13.6
*Mansonia humeralis*	2	6.3	-	-
*Mansonia indubitans*	1	3.1	3	5.1
*Mansonia pseudotitillans*	-	-	4	6.8
*Mansonia titillans*	8	25.0	18	30.5
Taxa (S)	5		6	
Abundance (N)	32		59	
Shannon (H’)	1.341		1.566	
Pielou (J’)	0.833		0.874	
Jaccard index (Ji)	0.429			

**Table 4:** Parameters and p-values from the Generalized Linear Model results with negative binomial distribution explaining the abundance of Culicidae tribes collected at the Sítio Recanto Preservar, municipality of Silva Jardim, state of Rio de Janeiro, Brazil, between May 2023 and December 2024. Predicted variables: species, seasonal period (dry, rainy), temperature, humidity and rainfall.

	Estimate	Std. Error	z-Value	Pr (>|z|)	Significance
(Intercept)	−11.205	0.5175	−2.165	0.0304	*
Species: *Coquillettidia fasciolata*	0.9038	0.5888	1.535	0.1248	
Species: *Coquillettidia venezuelensis*	−0.6994	0.6978	−1.002	0.3163	
Species: *Mansonia humeralis* ^1^	−17.300	0.8782	−1.970	0.0488	*
Species: *Mansonia indubitans*	−12.140	0.7715	−1.574	0.1156	
Species: *Mansonia pseudottillitans*	−12.886	0.7847	−1.642	0.1005	
Species: *Mansonia tillitans*	0.6744	0.5973	1.129	0.2589	
Seasonal period: Rainy ^2^	12.575	0.6006	2.094	0.0363	*
Temperature	0.2992	0.2920	1.025	0.3056	
Humidity	−0.1228	0.2674	−0.459	0.6459	
Rainfall	−0.1371	0.3186	−0.430	0.6669	

^1^ Difference from *Coquillettidia chrysonotum*. ^2^ Difference from Dry Season. Signif. codes: ‘*’ (1 asterisk): 0.05.

## Data Availability

All data supporting the findings of this study are available within the paper and its Supplementary Materials. Correspondence and requests for materials should be addressed to J.A.

## Supplementary Materials

The following supporting information can be downloaded at: https://www.mdpi.com/article/10.3390/tropicalmed11020039/s1, Table S1: Results of the Simper analysis (Similatity Percentages) for the contrast in abundance as a function of the CDC trap height (1.5 and 10 meters high) of the Mansoniini Diptera collected at Sítio Recanto Preservar, Silva Jardim, Rio de Janeiro, between May 2023 and December 2024; Table S2: Results of the Simper analysis (similarity percentages) for the contrast in abundance as a function of CDC trap height (1.5 and 10 meters high) of species of the Mansoniini tribe collected at Sítio Recanto Preservar, Silva Jardim, Rio de Janeiro, Brazil, between May 2023 and December 2024.
