# Supplementary material for: Seasonal Dynamics Versus Vertical Stratification of Mosquitoes (Diptera: Culicidae) in an Atlantic Forest Remnant, Brazil: A Focus on the Mansoniini Tribe
Source: Trop Med Infect Dis. 2026 Jan 30;11(2):39. doi: 10.3390/tropicalmed11020039 (PMC12945141; doi:10.3390/tropicalmed11020039)
Supplement: Supplementary file 1 [file tropicalmed-11-00039-s001.zip › tropicalmed-4055177-supplementary.pdf]

Supplementary Materials

Table S1. Results of the Simper analysis (Similatity Percentages) for the contrast in abundance as a function of the CDC trap height (1.5 and 10 meters high) of the Mansoniini Diptera collected at Sítio Recanto Preservar, Silva Jardim, Rio de Janeiro, between May 2023 and December 2024.

Species	average	sd	ratio	ava	avb	cumsum	contrib (%)	p
Cq. fasciolata	0.245	0.233	1.054	1.455	1.889	0.331	33.1	0.717
Ma. titillans	0.186	0.186	1.000	0.727	2.000	0.582	25.1	0.420
Cq. chrysonotum	0.136	0.171	0.792	0.636	0.778	0.765	18.3	0.464
Ma. pseudotitillans	0.053	0.124	0.432	0.273	0.111	0.838	7.3	0.864
Cq. venezuelensis	0.052	0.088	0.593	0.364	0.444	0.908	7.0	0.437
Ma. humeralis	0.038	0.103	0.367	0.182	0.000	0.959	5.1	0.567
Ma. indubitans	0.030	0.054	0.562	0.091	0.333	1.000	4.1	0.168

Table S2. Results of the Simper analysis (similarity percentages) for the contrast in abundance as a function of CDC trap height (1.5 and 10 meters high) of species of the Mansoniini tribe collected at Sítio Recanto Preservar, Silva Jardim, Rio de Janeiro, Brazil, between May 2023 and December 2024.

Species	average	sd	ratio	ava	avb	cumsum	contrib (%)	p
Cq. fasciolata	0.259	0.230	1.127	1.083	2.500	0.331	33.1	0.305
Ma. titillans	0.191	0.188	1.017	0.667	2.250	0.574	24.3	0.310
Cq. chrysonotum	0.116	0.150	0.775	0.667	0.750	0.723	14.9	0.977
Ma. pseudotitillans	0.087	0.148	0.586	0.000	0.500	0.833	11.0	0.002
Cq. venezuelensis	0.075	0.103	0.722	0.000	1.000	0.928	9.5	0.015
Ma. humeralis	0.028	0.047	0.595	0.083	0.375	0.964	3.6	0.435
Ma. indubitans	0.028	0.085	0.329	0.167	0.000	1.000	3.6	0.970
